# *Plasmodium falciparum* drug resistance in Angola

**DOI:** 10.1186/s12936-016-1122-z

**Published:** 2016-02-09

**Authors:** Cláudia Fançony, Miguel Brito, Jose Pedro Gil

**Affiliations:** Health Research Center of Angola (CISA, translated), Caxito, Angola; Lisbon School of Health Technology, Polytechnic Institute of Lisbon, Portugal, Lisbon, Portugal; Drug Resistance Unit, Division of Pharmacogenetics, Department of Physiology and Pharmacology, Karolinska Institute, Stockholm, Sweden

**Keywords:** Malaria, Drug efficacy and molecular markers

## Abstract

Facing chloroquine drug resistance, Angola promptly adopted artemisinin-based combination therapy as the first-line to treat malaria. Currently, the country aims to consolidate malaria control, while preparing for the elimination of the disease, along with others African countries in the region. However, the remarkable capacity of *Plasmodium* to develop drug resistance represents an alarming threat for those achievements. Herein, the available, but relatively scarce and dispersed, information on malaria drug resistance in Angola, is reviewed and discussed. The review aims to inform but also to encourage future research studies that monitor and update the information on anti-malarial drug efficacy and prevalence of molecular markers of drug resistance, key fields in the context and objectives of elimination.

## Background

*Plasmodium falciparum* malaria is one of the most important public health problems in Angola, with more than three million cases confirmed between 2000–2013 and 7300 attributed deaths in 2013 [1]. Accordingly, in 2004 the country adopted artemisinin-based combination therapy (ACT) as the first-line treatment to uncomplicated malaria cases, specifically artemether-lumefantrine (AL, Coartem^®^, Novartis, Basel), having reached full territory implementation between 2007–2008 [2]. Additionally, considerable efforts have been made by the Angolan authorities, which have set a five-fold increase in public financing for malaria control [3]. Thus, the country has also become a signatory of the Malaria Elimination Eight (E8) network in 2009, aiming to consolidate malaria control and prepare for the ambitious elimination of the disease in the West-Southern African countries [1, 4].

Highly efficient treatments (ACT), have been a key factor for the improvements on the global control of malaria, and will be cornerstone in the transition to the elimination phase. Unfortunately, the remarkable capacity of the parasite to develop drug resistance represents a threat for these objectives. In this context, the present knowledge of malaria drug resistance in Angola is herein reviewed.

### From quinine to chloroquine resistance

The first available anti-malarial drug in Angola, as in all the Western World, was quinine mono-therapy, introduced during the Portuguese colonial times. Albeit clinical failure with quinine therapies had been known for decades, there are no reliable reports of drug resistance when used in the country during the first decades of the 20th Century. This apparently sustained efficacy might be related to the limited deployment of the drug, partly due to its historical low supplies [5–7].

During the 1950s, few clinical trials were conducted in Huambo province (south Angola), testing the efficacy of pyrimethamine with weekly doses during 24 weeks, motivated by the low cost of this drug [8]. No evidence of resistance was documented, as all treated subjects were parasite free after the completion of the trial. In the same decade, chloroquine (CQ) was introduced in Angola, in line with the Global Malaria Eradication Programme and following the successful elimination of the disease in Portugal [9]. This event was the first significant malaria control effort targeting large sectors of the population.

In the late 1950s, CQ resistance emerged from foci in both South East Asia and South America. From the Asian focus, resistant *P. falciparum* progressively expanded in a western direction and by the start of the 1970s signs of possible CQ resistance were reported in the African continent [10–12]. The reports were unclear until the end of the decade, but unequivocal evidences of endemic CQ resistance were confirmed in Africa in 1979 [13, 14]. It is now assumed that CQ resistance then expanded in an east–west direction [15]. In 1984/1985, the first cases of CQ resistance were identified in Angola, particularly among European travellers [16, 17].

### In vivo drug resistance

#### Trials supporting the use of ACT

Efficacy clinical trials, recommended to be conducted every 2 years, constitute the gold standard for the monitoring of drug efficacy, identification of anti-malarial drug resistance and to guide drug policy. Such studies were not systematically performed in Angola for an extended period of time, due to social instability. Thereafter, in 2001, the Ministry of Health conducted a series of in vivo anti-malarial efficacy trials in several provinces. In two of the implementation sites (Huambo and Bié provinces) the follow-ups were extended to 28 days and ACT was tested in a population of children below 5 years of age [18, 19]. A detailed report was published, showing that CQ [25 mg/Kg, 3 day course, 10 mg/Kg/day, first 2 days, 5 mg/Kg on the last day (IDA, Netherland)] and sulfadoxine-pyrimethamine (SP) (25 mg/Kg sulfadoxine + 1.25 mg/Kg pyrimethamine, one dose) had low efficacy, justifying the presence of resistant parasites among the children infected [19]. Inter-site average clinical and parasitological failures were above 80 % for CQ and >30 % for SP, upon PCR-correction through *pfmsp1* and *pfmsp 2* analysis. Amodiaquine [AQ, 30 mg/Kg, 3 day course, 10 mg/Kg/day (Camoquin^®^, Park-Davis, Senegal)] was associated with about 20 % of failure rate. On the other hand, the two ACT formulations tested, artesunate (AS) + SP (artesunate 4 mg/Kg/day, 3 days + 25 mg/Kg sulfadoxine, 1.25 mg/Kg pyrimethamine 3 days) and artesunate-amodiaquine [ASAQ: AS, 4 mg/Kg/day, 3 days (Arsumax^®^, Sanofi Winthrop, France) + AQ, 10 mg/kg/day, 3 days] were highly successful, with cure rates of 98.5 % for both [19]. Nevertheless, the low efficacy of SP therapy motivated the authors to recommend ASAQ as a better option for the post-chloroquine upgrade of the Angolan national malaria programme. This Médecins sans Frontières driven study, was extended in the following year with the objective to compare ASAQ with the artemether-lumefantrine (AL) fixed combination (Coartem^®^, Novartis, Basel) [18]. Also performed in the Huambo Province, this small study demonstrated that both ASAQ or AL were highly efficacious and no PCR-corrected (*pfmsp1/2* + *gulp*) clinical failures were observed. It should be noted that others have demonstrated the occurrence of recrudescence 28 days after treatment initiation (especially with AL) [20–22]. Following this, Angola has (since 2007–2008) fully implemented ACT as the first-line mainstay for the national malaria control programme.

#### Recent suspicions of in vivo lumefantrine (LUM) resistance

In 2013, an ACT efficacy trial tested the efficacy of AL (20 mg artemether + 120 mg lumefantrine, 3 days) and the efficacy of a second generation ACT dihydroartemisinin-piperaquine (DHA-PPQ, Duo-cotecxin^®^; Beijing Holley-Cotec, China) in children under 5 years of age, in the Provinces of Zaire and Uíge [23]. DHA-PPQ showed extremely high efficacy, with no observed clinical failures (PCR-corrected), similar to results in other African locations [24]. The most impressive result was the relatively poor AL performance, with a final (corrected) efficacy below the WHO threshold for acceptable ACT efficacy (90 %) [1]. These results prompted a discussion regarding the possibility of AL resistance [25, 26]. On one hand, it should be taken into consideration that this result was mainly observed in one of the sites (Zaire Province) and involving a relatively low number of patients (n = 79). Importantly, as not all the AL doses were supervised, and no LUM blood levels were determined, questions rises if a substantial fraction of the observed clinical failures were due to lack of compliance, which would be consistent with increased cases of treatment failure in AL effectiveness trials [27]. Nevertheless, the results were robustly PCR-corrected, and a higher prevalence of *pfmdr1* 86N/184F/1246D genotypes, reported to be associated with LUM resistance, were documented among the recrudescence cases (when compared with the reinfections), suggestive of possible resistance [28]. The results of Plucinski et al. need to be followed up with new larger trials specifically designed for the detection of putative resistant infections [27].

In 2015, a small scale AL efficacy study, dispersed in 3 years, was performed in three different health centres of Luanda [29]. This research study also suggested a potential reduction in the AL PCR-corrected cure rates from the 2004 data (99 % towards 91.5 %) [29]. Despite intriguing, the limited size of the study, as well as the fact of being conducted in three different years and several different locations, recommends caution on the interpretation of those results. Even though it is open to discussion, the mere possibility of AL resistance emerging in Angola is worrisome enough, as this combination represents the cornerstone of malaria control in country.

#### Resistance to artemisinin derivatives

A recent case report, described a malaria patient returning from Luanda to a non-malarious region in Vietnam, showing a poor response to intravenous artesunate, as well as to a following dihydroartemisinin-piperaquine (Sigma-Tau pharmaceuticals, Gaithersburg, USA) treatment [30]. The patient was rescued by treatment with quinine-doxycycline. The very long clearance time of this infection (above 100 h) in the genetic environment of a wild type *k13*-*propeller* gene, along with the presence of multiple genotypes, an unusually slow response to all the used drugs and the lack of pharmacokinetic data, has raised questions about the possibility of patient factors justifying the clinical failure, including functional asplenia [31]. Concerns about the quality of the drug used were also raised. The authors argued that the low drug exposure would not justify the long persistence of high parasitaemia (>200,000 parasites/µL), while situation of severe asplenia would prolong the clearance of the parasites for even longer periods, irrespective of the drug used [32]. The rapid effect of the quinine + tetracycline rescue treatment supports this view. As for the raised issues on drug quality, the DHA-PPQ batches were verified directly by the manufacturer.

The case is essentially inconclusive, with the original authors considering that the critical importance of early detection of artemisinin resistance in Africa justifies high sensitivity surveillance, which can be more prone to false positive events than to clinical and epidemiologically more serious false negative situations. It is to note that Plucinski et al. did not find any evidence of a reduction of artemisinin derivative efficacy during their ACT efficacy clinical trial [23].

A compilation of available data on anti-malarial drug efficacy trials conducted in Angola is presented in Table 1.

**Table 1:** Efficacy of antimalarial drugs in Angola

Reference	Location	Drug therapy	Follow up	Results (between baseline and follow up)		
Cambournac et al. [8]	Province: Huambo Type of study: efficacy, case-control (placebo), probably partially randomized. Bind to the patient Sampling date: 1953 Participants: 200, mainly < 515 year old.	Pyrimethamine	24 weeks	All treated subjects were parasite free after the completion of the trial. 5.4 % of the participants in the control group (placebo) carried parasites at endpoint		
Suleimanov SD [62]	Province: Type of study: ex vivo survey Sampling date: 1991–1992 Participants: 105	CQ Fansidar Quinine-tetracycline		Resistance CQ: 61 % showed resistance Fansidar: 40 % showed resistance Quinine-tetracycline: 56 % were successfully cured Quinine-Delayed elimination of the parasites within 7 days of initiation of the therapy		
Guthmann et al. [19]	Provinces: Huambo and Bié Type of study: efficacy, partial randomization, with “absence of allocation concealment” Sampling date: 2002–2003 Participants: 619 children (240 Huambo and 379 Bié)	CQ-Huambo AQ-Huambo and Bié SP-Huambo and Bié AQAS-Bié SPAS-Bié	28 days	Failure rates CQ-84 % AQ-17 % in Huambo and 22 % in Bié SP: 25 % in Huambo and 39 % in Bié AQAS-1.2 % SPAS-1.2 %	Decreased anemia AQ-86 to 45 % SP–85 to 66 % in Huambo and 93 to 66 % in Bié AQAS–83 to 39 % SPAS-90 to 37 %	
Guthmann et al. [18]	Provinces: Huambo and Bié Type of study: randomized efficacy trial Sampling date: 2004 Participants: 137 children (6–59 months)	AL ASAQ	28 days	Reinfection AL-3.2 % ASAQ: 6.2 %	Decreased anemia AL: 54 to 13 % ASAQ: 53 to 16 %	Cure rates AL and ASAQ: 100 %
Kiaco et al. [29]	Province: Luanda Type of study: Interventional prospective cohort Sampling dates: 2011, 2012 and 2013 Participants 103 children and adults (6 months to 56 years; 37 from 2011, 33 from 2012 and 33 from 2013)	AL	28 days	Adequate clinical and Parasitological response: 90 % Global failure rate (before PCR correction): 9.7 % Samples With *pfmdr1* increased copy number + adequate clinical and Parasitological response: 92 %		Cure rate: 91 %
Plucinski et al. [23]	Province: Uíge and Zaire Type of study: open-label, nonrandomized Sampling date: 2013 Participants: 320 children (6–108 months)	AL DHA-PPQ	28 days	Treatment failure AL-7.5 % DHA-PPQ-0.3 % Adequate clinical and parasitological response AL: Zaire-88 % and Uíge-97 % DHA-PPQ: Zaire-100 % and Uíge-100 %		

#### Molecular markers of drug resistance

The first clinical trials by Guthmann et al. showed that the efficacy of all mono-therapies available in Angola was below the minimum benchmark adopted by the World Health Organization (WHO) [19, 33]. These findings motivated a number of studies in the following years investigating molecular markers of resistance in the country (Table 2).

**Table 2:** Molecular markers of antimalarial drug resistance in Angola

Reference	Study design	Gene	Main results					
Pinheiro et al. [55]	Province: Luanda Sampling data: unknown Number of participants: 15 Population: Travelers from Luanda (age unknown)	*Pfmdr1*	*Pfmdr1* 86: T-73 %* 1246: 0 %					
Pinheiro et al. [55]	Province: unknown Sampling data: unknown Number of participants: 9 from Angola Population: unknown	*pfdhfr*	*Pfdhfr* 108: S-11 %, N-11 % 108/51: NI-44 % 108/59: NR-11 % 108/51/59: NIR-22 %					
Kryger et al. [37]	Province: Bengo (Kifangondo) Sampling data: 1999 Number of participants: 168 (59 analyzed) Population: unknown	*pfcrt*	*pfcrt* 76:T-98 %					
Pimentel et al. [70]	Province: Luanda Sampling data: 2013/2014 Number of participants: 249 Population: Under 12 years	*pfcytb*	*Pfcytb* 268: 0 % of mutations associated to atovaquone-proguanil treatment failure					
Figueiredo et al. [39]	Province: Luanda Sampling data: unknown Number of participants: 245 Population: children between 1–16 years	*pfcrt* *pfmdr1* *pfdhfr* *pfdhps*	*pfcrt* 76: T-94 %, K-5.7 % KT-0.4 %	*pfmdr1* 86: Y-61 % N-29 % NY-10 %	*pfdhfr* 59: C-61 % R-21 % CR-19 %		*pfdhps* 540: K-88.3 % E-6.3 % KE-5.4 %	*Pfdhfr/pfdhps* (51/59/108)/ (437/540) Quintuple mutant: IRNGE-9 %
Menegon et al. [38]	Province-Úíge Sampling data-2004 Number of participants-66 Population-Children	*pfcrt* *pfmdr1* *pfdhfr* *pfdhps* *pfATPase6*	*pfcrt* 72/74–76: CIET-94 % CMNK-6 % SMNT-0 %	*pfmdr1* 86 Y-36 % N-32 % NY-32 %	*pfdhfr* 50C-100 % 51I-95 % 59R-36 % 108N-97 %	*pfdhps* 436A-52 % 437G-92 % 540K-100 % 581A-100 % 613A-100 %	*pfATPase6* 243Y-3 % 431K-24 % 402/771: VE-3 % 263/623/769: LAS-100 %	*Pfcrt* 76/ *pfmdr1*86/ *pfdhfr* 51,59,108/ *Pfdhps*436, 437 haplotypes: Sixfold mutated: 36 % Fivefold mutated: 33 % Sevenfold mutated: 14 %
Gama et al. [40]	Province: Luanda Sampling data: 2007 Number of participants: 114 Population: >18 years	*Pfcrt* *Pfmdr1*	*Pfcrt* 72-76: CVIET-13 % StctVMNT-57 % CVMNT-3.9 % CVMDT-2.9 %			Pfmdr1 86, 130, 184, 1034, 1042, 1109 and 1246: YEYSNVD-61 % NEFSNVD-11 % NEYSNVD-21 %		
Fortes et al. [64]	Provinces: Huambo, Cabinda, Uíge, Kwanza Norte and Malanje. Sampling data: unknown Number of participants: 452 Population: under five children	*pfdhfr* *pfdhps*	*pfdhfr* Total 51: I-90 %, NI-7.5 % 59: C-51 %, R-20 %, CR-29 % 108: N-99 % 51/59/108: IRN-25 %, ICN-72 % Malange 51: N-3.4 %, I-83 %, NI-14 %, 59: C-63 %, R-5.9 %, CR-32 %, 108: S-1.5 %, N-98 %, SN-0.5 % 51/59/108: IRN-3.9 %, ICN-91 % Kuanza Norte 51: N-2.1 %, I-95 %, NI-3 % 59: C-50 %, R-27 %, CR-24 % 108: S-0 %, N-100 %, SN-0 % 51/59/108: IRN-32 %, ICN-65 % Cabinda 51: N-0 %, I-99 %, NI-1.4 % 59: C-28 %, R-48 %, CR-24 %, 108: S-0 %, N-100 %, SN-0 % 51/59/108: IRN-64 %, ICN-36 % Úige 51: N-1.9 %, I-96 %, NI-1.9 % 59: C-36 %, R-28 %, CR-36 % 108: S-0 %, N-100 %, SN-0 % 51/59/108: IRN-41 %, ICN-56 % Huambo 51: N-0 %, I-96 %, NI-4.4 % 59: C-46 %, R-27 %, CR-27 % 108: S-0 %, N-100 %, SN-0 % 51/59/108: IRN-17 %, ICN-83 %			*pfdhps* Total 437: G-83 %, AG-11 % 540: K-87 % 437/540: GK-91 %, GE-3.2 %, AK-5.6 % Malange 437: A-7.7 %, G-75 %, AG-16 % 540: K-91 %, E-2.4 %, KE-6.3 % 437/540: GK-89 %, GE-3.1 %, AK-7.5 % Kuanza Norte 437: A-0 %, G-97 %, AG-3.1 % 540: K-96 %, E-2.1 %, KE-7.5 % 437/540: GK-95 %, GE-2.1 %, AK-3.2 % Cabinda 437: A-12 %, G-86 %, AG-1.5 % 540: K-83 %, E-4.5 %, KE-12 % 437/540: GK-88 %, GE-5.3 %, AK-7.0 % Úige 437: A-0 %, G-96 %, AG-3.7 % 540: K-98 %, E-1.9 %, KE-0 % 437/540: GK-98 %, GE-1.9 %, AK-0 % Huambo 437: A-13 %, G-38 %, AG-50 % 540: K-61 %, E-5.6 %, KE-33 % 437/540: GK-63 %, GE-13 %, AK-25 %		*pfdhfr/pfdhps* mix: Total ICN/GK-63 % IRN/GK-25 % Malange ICN/GK-81 % IRN/GK-3.9 % Kuanza-Norte ICN/GK-60 % IRN/GK-29 %) Cabinda ICN/GK-28 % IRN/GK-61 % Uíge ICN/GK-55 %, IRN/GK-45 % Huambo ICN/GK-25.0 % IRN/GK-25.0 %
Fancony et al. [46]	Province: Bengo Sampling data: 2010 Number of participants: 541 Population: children and adult their caretakers	*Pfcrt* *Pfmdr1*	*Pfcrt* 72–76: CVIET-38 % CVMNK-37 % SVMNK-0 % CVMNK/CVIET-25 %			*Pfmdr1* 86: N-68 %, Y-17 %, NY-15 % 184:Y-61 %, F-27 %, YF-12 % 1034, 1042 and 1246: SND-100 % 86/184/1034/1042/1246: YYSND-18 %, NFSND-34 %, NYSND-48 %		
Kaingona [65]	Province: Huíla Sampling data: unknown Number of participants: 110 Population: children and adult their caretakers	*pfdhfr* *pfdhps*	*pfdhfr* Total 108: N-98 % 59: R-65 % 51: I-47 % 164: L-2 % Single 108N-2 % Double mutations: 59/108: RN-48 % 51/108: IN-29 % Triple mutations 51/59/108: IRN-17 % 50/51/108 RIN-2 % 59/108/164: RNL-2 %			*pfdhp*s Total 437: G-96 % , A-4 % Single 437: G-88 % Double mutations 437/540: GE-8.3 % 436/437: A/F+G-4.2 % Triple mutations 436/437/540 SGK-88 % SGE-8 % AGK-4.5 %		
Ngane et al. [47]	Province: Benguela Sampling data: 2010/2011 Number of participants: 60 Population: under 15 years old	*pfcrt* *pfmdr1* *pfdhfr* *pfdhps*	*pfcrt* 72-76: CVMNK-11 % CVIET-89 %	*pfmdr1* 86, 184, 1034, 1042, 1246: NYSND-35 %, YYSND-48 % NFSND-11 % YFSND-3.7 % YYSNY-1.8 %		*pfdhfr* 16, 51, 59, 108, 164 ANCSI-3.4 % ANCNI-1.7 % AICNI-69 % ANRNI-5.2 % AIRNI-21 %.		*pfdhps* 436, 437, 540, 581, 613: SAKAA-13 % SAKAA-13 % SGKAA-60 % AGKAA-27 %
Plucinski et al. [23]	Province: Zaire and Uíge Sampling data: 2013 Number of participants: 25 Population: children	*pfmdr1* *Chr 10* *Chr 13* *pfk 13 propeller*	*pfmdr1* 86/184/1246: Uíge NFD-17 % NYD-50 % YYD/NFD-17 % YFD/YYD-17 % Zaire NFD-26 % NYD-32 % YYD-5.2 % YYD/NFD-5.2 % YFD/YYD-5.2 % NFD/YYD-5.2 % YYY/NYD-11 % NFY/NYD-5.2 % NYD/NFD-5.2 %	Chr 10 688956: Wild-type-100 %		Chr 13 1718319 Wild-type-100 %		*K13* Wild-type-100 %
Escobar et al. [73]	Province: Luanda, Malange and Kwanza Norte Sampling data: 2003–2010 Number of participants: 100 Population: unknown	*pfk13-propeller*	*K13* 471, 493, 539, 543, 575, 580 Total R471R-2 % R575R-1 % 493/539/543/580: HTTY-0 % Malange R471R-1 % R575R-1 % Kwanza Norte-0 % Luanda-R471R-1 %					
Kiaco et al. [29]	Province: Luanda Sampling dates: 2011, 2012 and 2013 Number of participants: 103 (37 from 2011, 33 from 2012 and 33 from 2013) Population: 6 months to 56 years old	*pfmdr1* *pfatp6* *pfk13*-propeller	*pfmdr1*86: N-73.4 % (68 % in 2011, 69.7 % in 2012 and 82 % in 2013) Y-18.1 % (21 % in 2011, 18 % in 2012 and 15 % in 2013) NY-8.5 % (11 % in 2011, 12 % in 2012 and 3 % in 2013) 1246 D-99 % (100 % in 2011, 97 % in 2012 and 100 % in 2013) Y-1 % (0 % in 2011, 3 % in 2012 and 0 % in 2013)			*pfatp6* 769 S-100 %		*K13* 493, 539, 543 and 580: Wild-type (YRIC)-100 %

*Only half of the samples are from Angola

##### *Pfcrt* (*Plasmodium falciparum* chloroquine resistance transporter)

The *pfcrt* K76T is strongly linked with in vitro leap increases in IC_50_ values and clinical failure of CQ canonical regimen (3 day, 25 mg/Kg) [34–36]. The first report on *pfcrt* mutations in Angola came from the analysis of a small study performed in 1999 among uncomplicated malaria patients in Kifangondo, near Luanda [37]. The 76T SNP was found in 51 out of the 52 analysed infections. This high prevalence was supported by a following larger study in Luanda, where *pfcrt* 76T was found in all the ca 250 studied samples, and in a subsequent smaller survey in the Uíge Province (with >90 % of the parasites carrying the 76T allele) [38, 39]. The latter study was done through PCR fragment direct sequencing, revealing the common 72–76 haplotype CVIET. In 2010, the study of a set of uncomplicated malaria patient samples, again from Luanda, held a surprise: the key amino acids 72–76 region of the gene was shown to be significantly polymorphic [40]. Contrarily to other African regions, where the Asian originated CVIET haplotype is almost exclusive, the CVMNT, CVINT and even a new one, CVMDT, were detected [15, 40]. More importantly, the most prevalent haplotype reported was the SVMNT, typical of South America regions [41, 42]. Albeit considered rare in Africa, this haplotype has been sporadically found in the East coast of the continent [43]. The importance of the Luanda finding lay in the fact that the *pfcrt* SVMNT has been robustly associated with decreased parasite response to amodiaquine in vivo and in vitro, and that ASAQ was until recently the second line treatment of uncomplicated malaria in the country [44, 45]. The finding of a significant prevalence of SVMNT haplotype-carrying infections could partly explain the observed relatively low efficacy of AQ monotherapy [17]. Neither way, a more comprehensive survey performed in Bengo province (neighbouring Luanda metropolitan region) failed to find this diversity, as the analysed parasites either carried the Asian CVIET haplotype or (in a smaller fraction) the globally frequent CQ sensitive CVMNK [46]. Similarly, a recent study performed in the southern province of Benguela, also has not detected other *pfcrt* haplotypes than CVIET and CVMNK [47]. Gama et al. [40] have hypothesized that their exceptional observations were due to imported parasite populations from South America, due to the intense commercial relation between Angola and Brazil and the highly cosmopolitan nature of Luanda. Although such explanation sounds interesting, it is not supported by the results concerning the analysis of the *pfmdr1* gene in the same parasites (see below). This aspect, and the previous detection of this haplotype in East Africa, recommends the surveillance of the potential emergence of *pfcrt* SVMNT carrying parasites, as proposed by Sá and Twu [48]. One positive aspect of the possible circulation of these haplotypes in Angola could be the fact that they have been associated with in vitro increased sensitivity to lumefantrine, when compared to the old World haplotype CVIET [49].

##### *Pfmdr1* (*Plasmodium falciparum* multidrug resistance 1)

*pfmdr1* was initially discovered during the quest for understanding CQ resistance [50]. Generally considered as a secondary factor, *pfmdr1* has proved to be a central gene in the ACT era. In Africa, soon after the introduction of this strategy, SNPs in this gene (N86Y, followed by D1246Y and Y184F) were consistently shown to be under selection pressure of AL (the N86/F184/D1246 haplotype) and ASAQ treatment (the 86Y/184Y/1246Y haplotype) [51–54]. In vitro studies confirmed those observations. More recently the importance of the *pfmdr1* alleles was reinforced through studies showing the increased capacity of N86/F184/D1246 parasites to invade patients with high lumefantrine blood levels upon AL treatment [28].

Upon a first small report, confirming the presence of CQ resistance associated to *pfmdr1* 86Y and 1246Y, a larger study conducted in Luanda showed that the large majority (ca. 90 %) of the parasite were carriers of the 86Y allele, similar to results from a smaller subsequent study in the Uíge province [38, 39, 55]. Gama et al. performed a more complete analysis, now also including the polymorphic positions Y184F, S1034C, N1042D, V1109I and D1246Y [40]. The authors struggled to PCR amplify fragments of this gene, with only 28 successful results. From this small sample, the frequency of the *pfmdr1* 86Y carriers was somewhat lower (ca. 60 %) as compared to the previously observed, while no mutations were detected in the amino acid 1246. Importantly, none of the parasites carried the characteristically New World 1034C or 1042D mutations. Such observation argues against the hypothesis that parasites harbouring *pfcrt* SVMNT haplotypes are exclusively imported from South American (see [40]).

In the Bengo province, the *pfmdr1* SNP frequencies were somewhat different from the previously observed in Luanda, as a dominance of the N86 allele, present in >80 % of the infections, was observed [46]. These differences were interpreted by the authors to a certain extent as the result of the withdrawal of CQ and the introduction of AL as the main anti-malarial in the public health system. To this possibility adds the contribution of different drug exposures between the populations of the more rural Bengo as compared with the capital, where private access to other anti-malarials (namely AQ, which selects the 86Y allele) is significantly facilitated.

*Pfmdr1* increased copy number has not been until recently identified in Angola, following the trend of its rarity in Africa [23, 56]. This scenario seems to be changing upon the report of a significant prevalence of this mutation in Luanda by the end of 2015 [29]. In a relatively small group of 101 successfully analysed AL treated patients, the amplification (2–3 copies) was detected in 13 subjects, the highest frequency ever registered in Africa [29]. Albeit that the presence of *pfmdr1* increased copy number was not associated with AL clinical failure, the known link between this mutation and multidrug resistance—including lumefantrine—justifies concern [57].

##### *Pfdhfr* (*Plasmodium falciparum dihydrofolate reductase*) and *pfdhps* (*Plasmodium falciparum dihydropteorate synthase*)

SP (Fansidar, Roche, Basel) was the ephemeral first solution after the progressive collapse of CQ in Africa. Unfortunately, and following the historical trend for antifolates, resistance against this combination rapidly rose [18]. Despite that SP resistance is spread in large regions of Africa, the drug is still a key mass drug administration tool for malaria intermittent preventive treatment (IPT) during pregnancy, as well as for seasonal malaria chemoprevention programmes.

SP resistance is mainly mediated by genetic mutations modifying its two specific targets, the dihydrofolate reductase and dihydropteorate synthase enzymes, that represent two key members of the folate synthesis pathway [58]. A set of five mutations—*pfdhfr*: N108**T**/N51**I**/C59**R** + *pfdhps* A437**G**/K540**E**—are linked to SP resistance in Africa, particularly when present as quintuple haplotype [59]. A sixth SNP (I164L), associated with further very high levels of resistance is rare in Africa, but has been occasionally detected [60, 61].

In 1991–1992, the WHO standard ex vivo tests were performed in infections under care at the Russian Hospital in Luanda. 40 % of the subjects carried SP resistance parasites [62]. The first molecular report also emerged from the capital, having been limited to the analysis of the *pfdhfr* C59R and K540E SNPs, assuming a previously observed high specificity and sensitivity of them (≥90 % for both) for detecting the presence of the quintuple haplotype [25, 39]. The combination of the two SNPs was only found in 9 % of the analysed samples. In a study performed also among uncomplicated malaria patients, this time in the province of Uige, a relatively high prevalence of triple *pfdhfr* mutants (~25 %) was found. All but one of these were actually quadruple mutants, as they also carried the *pfdhps* 437G allele. No analysis of the *pfdhps* 540 a.a. position was performed, precluding conclusions concerning the presence of the quintuple haplotype [38]. A new study, including the analysis of the five positions, was conducted by Gama et al. [63]. The *pfdhfr* triple mutant was present in 50 % of the samples. The PCR amplification of the *pfdhps* sequences was significantly less successful, but allowed the detection of the quintuple mutant. Finally, Fortes et al. collected peripheral blood samples from asymptomatic parasite carriers under 5 years of age, in a series of surveys conducted in five different provinces (Cabinda, Uíge, Kwanza Norte. Malange and Huambo) [64]. In total, ca. 450 subjects were enrolled, with the analysis being specifically focused on the five key mutations. The quintuple mutant was shown to exist in very low frequency (<1 %), with only two observations, one in Cabinda and another in Huambo. The *pfdhfr* triple haplotypes was anyway present in a quarter of the analysed infections, while the resistance associated to *pfdhps* 437G was the most frequent allele in this position (>80 %) [64]. Also, results from two studies conducted in the south, showed 48 and 29 % of *pfdhfr* double mutants and 17 % of triple mutants in Huíla province and 20 % of *pfdhfr* triple negative carriers and 0 % of the *pfdhfr/pfdhps* quintuple mutant (from 80 asymptomatic infections) in Balombo, Benguela Province (see Table 2) [47, 65].

The most important conclusion of this set of studies was formulated by Fortes et al. [64]. These authors alerted to the fact that the high frequencies of *pfdhfr* triple and the *pfdhps* 437G allele configure populations of parasites with a clear potential to rapidly evolve towards the quintuple resistant haplotype. This should be a matter of concern due to the remain importance of SP for intermittent preventive treatments, as well as the recently launched WHO seasonal malaria chemotherapy initiative, based in the mass drug administration of the SP/AQ combination [66]. The later has been associated with the ready selection of SP resistance-associated SNPs [67]. This information is relevant for Angola. Following the expected future reductions in transmission according to the Malaria Elimination Eight (E8) network objectives, some provinces—especially in the southern regions of the country—may likely be targeted for such mass administration programmes in the foreseable future.

*pf cytb* (*Plasmodium falciparum* cytochrome b).

The mitochondrial membrane-located cytochrome b is the specific target of atovaquone, the key component of Malarone^®^ (GSK, Brentford, UK). Malarone^®^ (250 mg atovaquone + 100 mg proguanil/tablet) is the most used prophylactic anti-malarial among travellers. In a similar trend as with SP, the parasite develops resistance to this drug by modifying its target. Mutations on *cytb* codon 268 (T268S/G) have been consistently associated with Malarone^®^ clinical failure, since the early days of the launch of this drug [68, 69]. An hospital-based PCR–RFLP study, performed in 249 malaria patients, in Luanda during 2003–2004, reported that all infections analysed were found to carry wild-type (sensitive) parasites for this a.a. position [70].

*pfatp6 [Plasmodium falciparum Ca(2*+*)*−*ATPase]* and *pf kelch13 (Plasmodium falciparum Kelch13 propeller* gene*)*

Both *pf*ATP6, as a potential target of artemisinin, and the Kelch 13 propeller cytoplasmic protein, as a mediator of response against artemisinin action, have been proposed as markers of *P. falciparum* artemisinin resistance. In *pf*ATP6, the S769N SNP was associated with significant increases in ex vivo determined IC_50_s in South America [71]. Diversity in the Kelch 13, particularly the C580Y SNP, has been linked with the phenotype of increased time of parasite clearance upon artemisinin-based therapies, in SE Asia [72].

In the only report mentioning *pfatp6*, the H243Y and the E431K SNPs were found to be present, the former being relatively frequent (~25 %). At the critical 769 position, only the wild type (S769) allele was observed in the set of analysed samples. As for the K13-propeller gene polymorphisms, a recent study have performed a small survey which consisted of 100 uncomplicated malaria patients from before and after the introduction of ACT in the country [73]. All sequences found were wild-type, with the exception of two synonymous SNPs, R471R and R575R. Those results indicate the low genetic variability of the gene in these regions, as well as the apparent lack of selective pressure from the introduction of ACT in the country. The low diversity observed is similar to the observed in the Zaire and Uíge provinces, where all the analysed parasites carried wild-type *k13*-*propeller* genes [58].

## Conclusions

### A number of knowledge gaps

In retrospect, the number of anti-malarial clinical trials performed in Angola has been meagre compared with the public health magnitude of this disease in the country. Additionally, the majority of the molecular studies were focused around Luanda. With its large territorial size, it would be expected that significant variation in the characteristics of the parasite populations may occur throughout its extensive geography.

Meanwhile, the recent increase in investment towards malaria control, expressed to a large extent in the introduction of effective ACT, has led to a significant decrease in the incidence of malaria in Angola in the last 5 years [1]. The country has the ambitious objective of reaching pre-elimination status during the next 5 years and, after decades of civil war, the country has reached a state of social stability compatible with such objectives. With its ample resources, and a relatively small population (comparatively to the standards of large African countries), Angola has the potential of being up to this challenge. However, such a goal still demands ground studies to answer a stream of key questions. Is the efficacy of lumefantrine really decreasing? What is the effectiveness of the other available formulations of ACT in Angola? With the pressure exerted by AL in the last years, is ASAQ now an even better alternative? Was the detection of *pfcrt* SVMNT a truly rare event or is the haplotype actually present in the country? Is the recently detected *pfmdr1* increased copy number also an unusual event, or should it be cause for concern?

These and other questions need to be answered in order to provide the local authorities with hard evidence, essential to support future key decisions towards the objective of elimination.
